# Humble Behaviorism Redux

**DOI:** 10.1007/s42822-022-00092-4

**Published:** 2022-03-25

**Authors:** Megan S. Kirby, Trina D. Spencer, Shane T. Spiker

**Affiliations:** 1 Department of Child and Family Studies, University of South Florida, 13301 Bruce B. Downs Blvd. MHC, Tampa, FL 1702 USA; 2 Mary Lou Fulton Teachers College, Arizona State University, Tempe, AZ USA

**Keywords:** Cultural humility, Humble behaviorism, Behavior analysis, Cultural reciprocity

## Abstract

The need to bring behavior analysis to scale is no more obvious or urgent than now. Collaboration between behavior analysts and healthcare workers, educators, policymakers, mental health clinicians, social workers, and so many other professionals is critical to reaching under-resourced and traditionally marginalized populations. First, however, interprofessional collaboration must be adopted widely and reinforced within the behavior analytic community. Disciplinary centrism and hubris pose barriers to effective interprofessional collaboration, leading one to assume the position that practitioners of the same discipline are better trained and smarter than those of a different field. However, cultural humility (Wright, *Behavior Analysis in Practice, 12*(4), 805–809, 2019) is an alternative to disciplinary centrism that allows professionals to retain identities born of cultural histories and training (Pecukonis, *Journal of Teaching in Social Work, 40*(3), 211–220, 2020). Furthermore, cultural reciprocity is a process of self-observation and collaborative inquiry that involves questioning one’s own assumptions and forces individuals (and professions) to confront the contradictions between their values and their practices (Kalyanpur & Harry, 1999). In this paper, we revisit the call for Humble Behaviorism first made by Alan Neuringer in 1991 and the recommendations of fellow behavior analysts since. Specifically, we introduce a framework of cultural reciprocity to guide humble behaviorists as they acquire behaviors necessary to establish and maintain productive interprofessional relationships. We encourage them to act on their ethical and moral duties to address social problems of global concern and bring behavior analysis to scale.

The need to bring behavior analysis to scale is no more obvious or urgent than now. In 2018, man-made or natural disasters and conflicts resulted in the displacement of over 40 million children around the world (Bothe et al., 2018). Such experiences can have detrimental effects on childhood development. For example, compared to regional averages pre-disaster, children affected by Hurricane Katrina were 11 to 15% more likely to engage in aggression, self-injury, have learning difficulties, and experience anxiety and/or post-traumatic stress three years after the event (Mclaughlin et al., 2009). Beyond natural disasters and conflicts, socio-economic status and race predict who has access to high-quality education in the United States (U.S.) and who is likely to experience incarceration in their lifetime (Annie E. Casey Foundation, AECF, 2017; Russo et al., 2017). More than one-third of U.S. school children are not proficient in basic reading skills by the end of fourth grade (National Center for Education Statistics, 2019). Approximately one quarter of students who drop out of high school read below grade-level, and African American and Hispanic students account for more than 60% of students who are unable to read proficiently and eventually drop out of school (AECF, 2017). These data reveal an intense need for improved services in traditionally marginalized and under-resourced communities and across the globe. Collaboration between behavior analysts, healthcare workers, educators, policymakers, mental health clinicians, social workers, and so many other professionals is critical to making evidence-based interventions accessible to vulnerable and deserving populations. However, interprofessional collaborative behaviors must first be widely adopted and reinforced by the behavior analytic community.

Scientists, including behavior analysts, know of interventions that can solve or ameliorate much of the suffering we encounter in our lives, directly or indirectly. Examples of such interventions include wearing seatbelts to reduce vehicular accident casualties (e.g., Geller et al., 1989) and wearing face masks to mitigate the transmission of COVID-19 (e.g., Abaluck et al., 2021; Pennington et al., 2021; Sivaraman et al., 2021). Scientists have also relied on behavioral science to design interventions that improve community recycling practices (e.g., Jang et al., 2020) and replace single-use plastics with reusable or compostable materials (e.g., Jia et al., 2019). Although behavior analysis may be less likely than other sciences to gain front-page recognition, slow uptake of evidence-based interventions is not unique to the field. Gambrill wrote, “Many objections to ABA are related to misunderstandings of science” (2012, p. 126), suggesting that contemporary society does not readily appreciate science in general. As a result, the dissemination of interventions derived from behavior analysis must compete with those without a scientific basis. It is already challenging for scientists to promote the adoption of facts, but behavior analysts’ reputation for engaging in prideful practices may further impede the scalability of behavior analysis (Freedman, 2016; Poling, 2010). If we are to achieve greater acceptance and adoption of behavior analysis, we cannot afford any amount of hubris.

In his 1991 paper, *Humble Behaviorism*, Allen Neuringer encouraged behavior analysts to adopt a position of humility as they practice among non-behavioral colleagues for the benefit of consumers. He suggests “If behaviorists were more humble, their effectiveness as scientists would increase” (p. 1). In the new Behavior Analyst Certification Board (BACB) Ethics Code, behavior analysis returns to this sentiment. Specifically, Codes 2.10 and 3.06 make it clear that behavior analysts are expected to collaborate and consult with colleagues to serve the best interests of their clients (BACB, 2020). One implication of this code revision is that behavior analysts can, and are encouraged to, promote the science of behavior through successful diffusion of interventions and positive interpersonal interactions during interprofessional teaming.

True adoption of humble behaviors may require behavior analysts’ and the organizations that train them to make a shift—one that questions current knowledge about, attitudes toward, and practices related to interprofessional collaboration. Therefore, in this paper, we revisit the call for Humble Behaviorism first made by Alan Neuringer (1991) and responses to the article since (see Volume 14, Issue 1 of *The Behavior Analyst* printed in 1991). We introduce several contemporary concepts related to interprofessional collaboration that, when understood, can facilitate the evolution of humble behaviorism. As current and future humble behaviorists sharpen their interprofessional behaviors, they will be in a better position to act on their ethical and moral responsibilities to address social problems of global concern and reach diverse and disenfranchised communities. To achieve humility, however, it is critical to recognize our greatest obstacle—disciplinary centrism.

## Disciplinary Centrism

Forceful dissemination, unwillingness to compromise, and poor communication with colleagues obstruct the scalability of behavior analysis. Such behaviors are often the result of a disciplinary-centric attitude. Disciplinary centrism is the belief that one’s own discipline is far superior to others and as a result, its practitioners are smarter and better trained (Pecukonis, 2020). Acceptable fervor and zeal may have motivated the establishment and maintenance of behavior analysis as an independent discipline, separating it from its American Psychological Association roots (Green, 1991; Thyer, 2015). However, when behavior analysts promote themselves and the science of behavior with a pride so exclusive and superior, they can offend the very people who may otherwise benefit from or advocate for behavior analytic practices (e.g., clients, families, colleagues, and society). Moreover, well-intended professional pride can result in our defiance of public opinion, such as the refusal to modify our language (e.g., Becirevic et al., 2016; Critchfield, 2017; Critchfield et al., 2017; Critchfield & Doepke, 2018; Foxx, 1996).

Disciplinary centrism within our field can also result in claims that programs designed by non-behavior analysts are unscientific and not supported by evidence when such practices do not readily fit within our behavior analytic model (e.g., Leaf et al., 2016; Leaf et al., 2018). For example, in a critique of Social Thinking®, a social skills program created by Michelle Garcia Winner and commonly used by speech and language pathologists (SLPs), Leaf et al. (2016) stated, “behavior analysts should not implement, recommend, or endorse Social Thinking®; doing so would violate the ethical guidelines described by the BACB®…These violations could result in disciplinary action against a certified behavior analyst” (p. 157). Furthermore, the title of the Leaf et al. (2016) article implied that the program was a pseudoscience. As a result, numerous behavior analysts may question the ethics of collaborating with “pseudoscientists” using the program (e.g., Long, 2017) or fear retribution from their peers if they do. While the actual rates of reports made to the BACB by fellow behavior analysts are low^1^ (BACB, 2018), the public threat of “disciplinary action” is visceral. Hubris is at the core of the idea that if an intervention wasn’t designed by a behavior analyst it is not scientific nor supported by evidence.

Unfortunately, behavior analysts who practice from a position of disciplinary centrism assume they sufficiently understand the beliefs, values, knowledge, and skills of other professionals and categorize them as “non-behaviorists.” The *us against them* bias blinds behavior analysts to the scientific and professional contributions of other disciplines. Biases can also motivate behavior analysts to create rules based on limited or false knowledge regarding the scope of other professionals’ competencies and practices (Belisle, 2020). Behavior analysts are not the only professional group susceptible to disciplinary centrism, obviously. However, due to the universal nature of the science, behavior analysts’ scopes of practice overlap with not just one or two other professions, but many. Thus, the need for behavior analysts to consider and be cautious of potential disciplinary centric attitudes is extensive.

If not corrected, hubris could lead behavior analysis into isolation, underground, or dissolution. Disciplinary centrism may stunt our science and the progression of our field. It threatens our survival (Poling, 2010). Over 30 years ago, Neuringer wrote, “There is much overlap between the ‘field’ of behavioral research and other areas…If humble behavioral practices yield scientific and social progress, they can survive the test of time” (1991, p. 11). The sentiment continues to ring true today. The progress of behavior science has been remarkable, but behavior analysis severely needs social progress.

The lack of humility demonstrated by a few behavior analysts has motivated the production of several publications to facilitate cross-disciplinary understanding (e.g., ABAI, 2020; Bowman et al., 2021; Brodhead, 2015; Cirincione-Ulezi, 2021; Kelly & Tincani, 2013; Koenig & Gerenser, 2006; LaFrance et al., 2019; Slim & Reuter-Yuill, 2021). We appreciate and celebrate our colleagues’ critical contributions to this discussion, but this topic has not yet been exhausted. Superb science skills, characteristic of behavior analysts, are generally underappreciated by society, but the “soft skills” of interpersonal communication, self-reflection, and compromise are in high demand. Until behavior analysts are known for their professional humility and exceptional collaboration skills, there is work to be done. This is especially true when we consider that almost an entire issue of *The Behavior Analyst* was dedicated to responses to Neuringer’s Humble Behaviorism paper, yet many professional behavior analysts have never heard of it (Cirincione-Ulezi, 2021). In 1991, Neuringer argued that “A humble stance with regard to other disciplines—asking for help in solving our problems—may, in the long run, serve all better than a continuation of the ‘you’re wrong/I’m right’ battles” (p. 11). We agree with Neuringer that interdependence with other professions, not independence from them, strengthens both the science and practice of behavior analysis. Therefore, we argue that the future of behavior analysis depends on the humble behaviorists’ ability to move beyond disciplinary power struggles and actively seek to bring about positive change in our world through strategic interprofessional collaboration.

## Interprofessional Collaboration Competencies

**Fig. 1:**
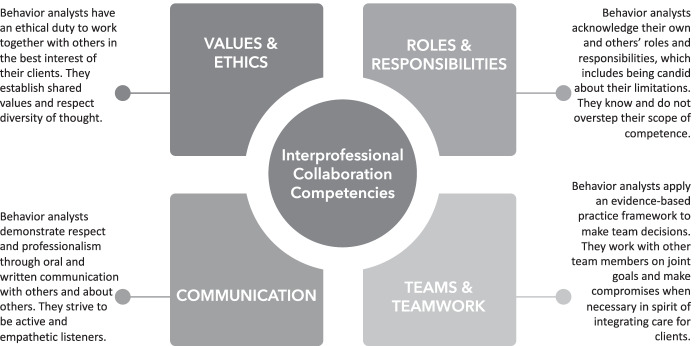
Interprofessional collaboration competencies, adapted from Interprofessional Education Collaborative (2016)

The World Health Organization (WHO) has been creating and disseminating information about interprofessional collaboration for many years (e.g., Gilbert et al., 2010). Other health professions (i.e., nursing, public health, occupational therapy, speech-language pathology and audiology, and social work) quickly adopted their framework and competencies. As many behavior analysts practice within the health arena, it is prudent to acknowledge the guidance provided by the WHO and to build upon their well-established foundation. Several of our peers have started this conversation (e.g., Bowman et al., 2021; Slim & Reuter-Yuill, 2021), but our field has yet to adopt the WHO interprofessional practice (IPP) framework. Capitalizing on technology that already exists, we briefly outline the core competencies put forth by the Interprofessional Education Collaborative (IPEC, 2016) and discuss how they relate to humble behaviorists (see ABAI, 2020 for another source) in the following sections. The practice of humble behaviorism will require behavior analysts to successfully collaborate with other professionals. The practice of humble behaviorism will also demand that behavior analysts demonstrate competencies in four key areas: (a) Teams and Teamwork, (b) Roles and Responsibilities, (c) Values and Ethics, and (d) Communication (see Fig. 1).

## Teams and Teamwork

The most efficient way for professionals from our young discipline to learn how to tackle large-scale problems familiar to more established disciplines (e.g., anthropology, biology, and medicine) is to embrace interprofessional collaboration. LaFrance et al. (2019) describe cohesive collaboration (or teaming) as “…multidisciplinary and transdisciplinary work, where professionals from different disciplines work together to identify goals, assess progress, and even cotreat” (p. 721). In education and community settings, it is common for individuals with developmental and/or intellectual disabilities to have multiple professionals implementing various interventions designed to improve their overall quality of life (Koenig & Gerenser, 2006; Monz et al., 2019; Pennington et al., 2016; Watson, 2016). For practitioners involved the treatment of autistic clients, Bowman et al. (2021) provide a set of core interprofessional collaboration standards. While there are many successful examples of interprofessional collaboration in this service arena, there have also been some missteps (e.g., Leaf et al., 2016; Rekers & Lovaas, 1974). For example, a brief search on various social media platforms can yield multiple examples of non-collaborative repertoires. Within major Facebook groups, we can see statements like “an SLP isn’t really necessary if the behavior analyst supervising the case is well versed in [Skinner’s] analysis of behavior.” In a podcast episode, behavior analysts support responding to individuals who criticize autism treatment approaches by telling them to, “Get over yourselves!” (Leaf & Cihon, 2020). Within these types of forums, we can directly observe public discourse that is often combative, non-collaborative, and even harmful to marginalized and vulnerable populations. Common discussions on public forums such as Reddit indicate that there is a collaboration and humility problem among behavior analysts. While we do not interpret anecdotal reports as truth, the commonality of these comments and many others suggests that there is some validity to these claims about the weak interprofessional repertoires of some behavior analysts.

In addition to the interprofessional challenges noted above, we see other troubling examples of disciplinary centrism within the behavior analytic community. In a presentation at the Florida Behavior Analysis Association conference in 2021, a senior leader who claims expertise in ethics stated that the field of behavior analysis is inherently unbiased, and that the primary cultural bias that behavior analysts experience stem from the families we serve, not from behavior analysts themselves. Within the same time frame, attacks on the legitimacy of culture also took place within the Teaching Behavior Analysis listserv (e.g., Brandon, 2021), further bolstering the reality of behavior analytic hubris.

As we work to redirect missteps and prevent further hubris, there is a need to expand our thinking about interprofessional work. Many behavior analysts expect to work alongside other healthcare professionals when practicing in outpatient or educational contexts; however, the potential application of behavior analysis extends far beyond challenging behavior and communication therapy. As we establish relationships with other scientists in arenas such as biotechnology, child welfare, criminal justice, business, safety, and climate change (to name a few), we should be preparing our early career professionals to work with, not just alongside, colleagues from different fields. In behavior analytic training programs, programmatic or departmental silos are often established and maintained. When students of behavior analysis are trained in a siloed model, they receive little to no instruction or practice in interprofessional collaboration and teaming (Brodhead, 2015; Kelly & Tincani, 2013). Interprofessional education (IPE) is when students from two or more disciplines learn about, from, and with each other (Gilbert et al., 2010). The primary purpose of IPE is to promote effective collaboration skills during formative stages of professionals’ education. IPE is largely missing in behavior analytic training programs, which likely contributes to the expression of disciplinary-centric attitudes when newly certified professionals are released into IPP contexts. To align ourselves with other professions (LaFrance et al., 2019) and to become known for humble and effective collaboration, we need to integrate IPE into our curricula and training programs and within our competence standards (Bowman et al., 2021; Callahan et al., 2019; Chadwell et al., 2018; Slim & Reuter-Yuill, 2021; St Peter, 2013).

## Roles and Responsibilities

In a team, each member contributes specialty knowledge and unique expertise. Starting from a position of humility, behavior analysts who collaborate effectively recognize themselves as a part of a larger system of services and supports designed to achieve a shared objective. Each team member has a specific role and a set of responsibilities that, in theory, complement the other team members’ responsibilities. It is the integration of these diverse roles and responsibilities that leads to superior care, both in terms of overcoming barriers to service delivery and enhancing consumer outcomes (Reeves et al., 2013). Humble behaviorists understand the difference between their scope of practice and their scope of competence and bring this distinction to bear when teaming. Brodhead et al. (2018) reminds us that scope of practice “refers to the range of activities in which members of a profession are authorized to engage, by virtue of holding a credential or license” (p. 425). In contrast, scope of competence is “the range of professional activities of the individual practitioner that are performed at a level that is deemed proficient” (Brodhead et al., 2018, p. 425). The areas where different professionals’ scopes of practice overlap can be exposed incidentally. However, when the exposure occurs deliberately, additional advantages are possible (MacDonald et al., 2010). For example, when team members explicitly share their areas of competence and educate each other on their scopes of practice, everyone on the team learns about the others’ professions. Frank conversations among team members can minimize confusion about individual roles and shared responsibilities. Furthermore, professional identities can be retained and respected through skilled negotiation of overlapping scopes (McNeil et al., 2013; Pecukonis, 2014).

In addition to acknowledging their own and others’ roles and responsibilities, humble behaviorists are candid about their limitations. As certification and graduate degrees in behavior analysis do not equate to content expertise (unless the content is behavior analysis), behavior analysts must rely on the experience and knowledge of content experts (e.g., speech-language pathologists, occupational therapists) to serve their clients properly. The practice of humble behaviorism requires that professionals do not overstep their scope of competence and never practice beyond their authorization. It is imperative that all behavior analysts model appropriate within-scope practice while helping their colleagues understand the porous boundaries. Misunderstandings about behavior analysts’ scopes of practice and competence are extremely common and are a frequent source of complaints about practicing behavior analysts (Hott et al., 2020; Volkers, 2020). Therefore, behavior analysts must be truthful and forthcoming about the limits of their competence and humble enough to support professionals who are more competent to assume a specific role or responsibility.

Similarly, behavior analysts should not arbitrarily restrict who can use the science of behavior (Brodhead et al., 2018). Certainly, professional behavior analysts do not own the principles of the science and are not the only practitioners qualified and entitled to use them; yet such sentiments exist. Principles of behavior have been and continue to be applied in a variety of disciplines, to include but not limited to sustainability and environmentally significant behavior change (e.g., Alavosius & Mattaini, 2011; Stern, 2000), education (e.g., Grisham-Brown & Hemmeter, 2017; Horner et al., 2005; Shepley & Grisham-Brown, 2018), social work (e.g., Clark et al., 2008; Kessler & Greene, 1999), psychology (e.g., Buchanan & Fisher, 2002; Dillenburger & Keenan, 2001; Friman et al., 1998; Weil et al., 2011), speech and language pathology (e.g., Esch & Forbes, 2017; Goldstein, 2002; Koenig & Gerenser, 2006), and nursing (e.g., Anbro et al., 2020). Behavior analysts readily use matrix training (Goldstein, 1983; Pauwels et al., 2015), but it was not originally developed by one. Goldstein is a speech-language pathologist who learned about and applied recombinatory generalization to promote new language and novel responses in his clinical practice. Behavior analysts do not own or control the science of behavior; if it is indeed a science, it applies universally. Universal application does not mean that behavior analysts have “an unconstrained scope of practice or an unlimited scope of competence” (ABAI, 2020, p. 2). It means that with competence (see Brodhead et al., 2018), any professional regardless of discipline can use it to bring about meaningful outcomes within their practice (Starry, 2016; White et al., 2018).

## Values and Ethics

The ethics of collaboration are central to the evidence-based practice of behavior analysis (Slocum et al., 2014). Humble behaviorists recognize that the practice of behavior analysis is founded on the same fundamental principles of ethics (e.g., *benevolence* and *do no harm*) as all other human service professionals (Contreras et al., 2021; Rosenberg & Schwartz, 2018). Likewise, behavior analysts believe that ethical decisions are made through the integration of the best available evidence, clinical expertise, and client and family preferences and context (BACB, 2020; Contreras et al., 2021; Rosenberg & Schwartz, 2018; Sackett et al., 1996; Slocum et al., 2014; Spencer et al., 2012). As all health professionals are charged with engaging in evidence-based practice (or medicine), it is the common ground upon which all team decisions are processed (Cox, 2012). Adopting the same definition of evidence-based practice as other health professions (Slocum et al., 2014) puts behavior analysts in a humble stance, and readies them for teaming. Being a part of an interprofessional team means that behavior analysts should strive to uphold the values, goals, and decisions made by the team, as is their ethical responsibility (BACB, 2020; Contreras et al., 2021; Cox, 2012). By honoring shared values and evidence-based processes, behavior analysts demonstrate their ability to be team players, which in turn promotes a favorable impression of behavior analysis.

While behavior analysts are likely to favor the evidence that supports their own assessment and treatment recommendations, humble behaviorists acknowledge that other disciplines such as occupational therapy and speech-language pathology have their own evidence. Their evidence is included in the concept of *best available evidence* and deserves equal consideration with respect to quality, quantity, and relevance (Slocum et al., 2012). By becoming acquainted with the research evidence of other professions, behavior analysts can expand the number of sources used to support their knowledge of best available evidence. Additionally, finding commonalities across disciplines in the varied literature can lead to fruitful conversations about the best evidence from which to draw practice recommendations (Morris, 2014). Normand et al. (2021) used the research of Michie et al. (2013) to describe similarities in health research and behavior analytic taxonomies that can lead to improved translation and further collaboration between disciplines. We posit that greater understanding of an interprofessional colleague’s research evidence promotes mutual respect and leads to improvements in patient or client care. Regardless of behavior analysts’ depth of cross-discipline knowledge, most professions hold progress monitoring as a critical element of evidence-based practice (Higginbotham & Satchidanand, 2019; Spencer et al., 2012). Guidance from Brodhead (2015) can support the use of progress monitoring to evaluate team decisions and implementation of non-behavioral practices. Likewise, progress monitoring data can facilitate communication within the team and reduce the personalization of opinions.

## Communication

The final IPEC competency, effective oral and written communication, permeates all interprofessional interactions. When behavior analysts are trained in a culture of disciplinary centrism, they acquire the communication style reinforced and maintained by that community. However, when the community shifts to an IPP context, listeners often punish (or avoid) behavior analysts for using the jargon-rich communication style they acquired in graduate school. The rigid use of that communication style may be abrasive and offensive to behavior analysts’ colleagues. Research has shown behavior analytic jargon such as *discrimination*, *chaining*, *punishment,* and *operation* have negative connotations and are often considered to evoke “unpleasant” feelings (Becirevic et al., 2016; Critchfield et al., 2017). Over 60% of our jargon is associated with negative emotions of English speakers, perhaps because historically, the words are associated with aversive social constructs; to the general listener, *chaining* and *operation* are terms closely associated with *bondage* and *surgery*, respectively (Critchfield et al., 2017). In contrast, humble behaviorists can strive to translate their off-putting terminology into friendlier terms and use lay definitions to avoid being misunderstood by teammates. It is also recommended that behavior analysts learn the basic terms and constructs that reflect their colleagues’ theories and guide their practices (Cox et al., 2018), a skill set that can serve as an establishing operation for bidirectional translation. As Claire St. Peter wrote, “I needed the help of specialists who were fluent in issues important to, and the language of, my target population” (2013, p. 156). Successful marketing of behavior analysis requires a respected audience, which includes current and future consumers and colleagues (e.g., Friman, 2010, 2014; Reed, 2014; Schlinger, 2014; Schneider, 2012). If people have a better understanding of the science and practice of behavior analysis, even if they do not use behavior analytic precision, they will know when to call upon us.

## Cultural Humility and Cultural Reciprocity

Having discussed the need to avoid disciplinary centrism and competencies integral to IPP, we now offer a single recommendation. To be effective collaborators, humble behaviorists can regard professional differences as cultural differences and embrace them. That’s it—cultural diversity is the key. Behavior analysts have made noteworthy strides in understanding and accepting culture as a behavioral determinant (Couto, 2019; Glenn, 1989, 2004; Malott & Glenn, 2006; Miller et al., 2019; Soares et al., 2019). The dawn of the new ABAI *Culturo-Behavior Science for a Better World* conference foreshadows an exciting future within our field, which emerged alongside enhanced cultural considerations embedded in the new Ethics Code (BACB, 2020). While we are not the first to suggest behavior analysis is a culture of its own, we argue that understanding and accepting cultural differences is central to effective interprofessional collaboration.

Sugai et al. (2012) defines culture as “the extent to which a group of individuals engage in overt and verbal behavior reflecting shared behavioral learning histories, serving to differentiate the group from other groups, and predicting how individuals within the group act in specific setting conditions” (p. 200). Although most readily understood in relation to racial and ethnic diversity, it also applies to the cultures of individual professions. For example, speech-language pathologists and occupational therapists receive two to three years of graduate training and supervised field experience to learn their vernacular, theories, and practices. The length of their learning histories approximates those of most professional behavior analysts (LaFrance et al., 2019), but the differences in their learning histories serve to separate the groups and predict how professionals in one group will act compared to another. Each group of professionals, despite vast within-group variation, engage in “a collection of common verbal and overt behaviors that are learned and maintained by a set of similar social and environmental contingencies” (Sugai et al., 2012; p. 200).

**Fig. 2:**
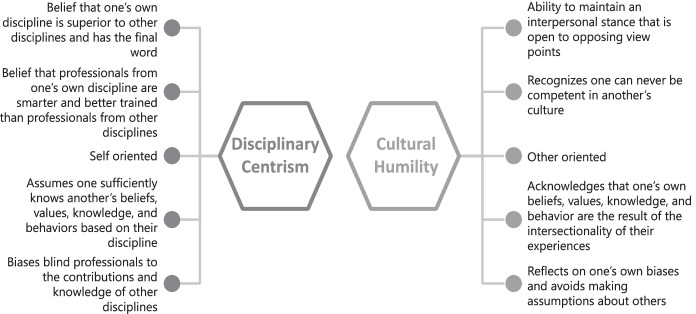
Comparison of disciplinary centrism and cultural humility

Cultural humility is the ability to maintain an interpersonal stance that is open to opposing viewpoints. It demands lifelong learning and a commitment to the disruption of power imbalances (Tervalon & Murray-Garcia, 1998). Embracing the socio-cultural movement within behavior analysis, Wright (2019) introduced the concept of cultural humility to behavior analysts based on literature from the fields of social work and other health professions (e.g., Fisher-Borne et al., 2015; Foronda et al., 2016; Zhang et al., 2021). We consider cultural humility to be the remedy for disciplinary centrism and have outlined point-by-point comparisons of the two concepts in Fig. 2. From a position of cultural humility, behavior analysts acknowledge that one’s own and others’ beliefs, values, knowledge, and behaviors are born of the intersectionality of multiple cultural identities related to race, ethnicity, sexual identity, religion, gender, disability, education, politics, etc. Importantly, the term cultural humility replaces cultural competence because it is unrealistic to be competent in another’s culture (Fisher-Borne et al., 2015; Wright, 2019). Furthermore, Wright contends that if behavior analysis “is going to expand its influence and ensure equal access, critical self-reflection and behavior change are necessary” (p. 808). We agree.

**Fig. 3:**
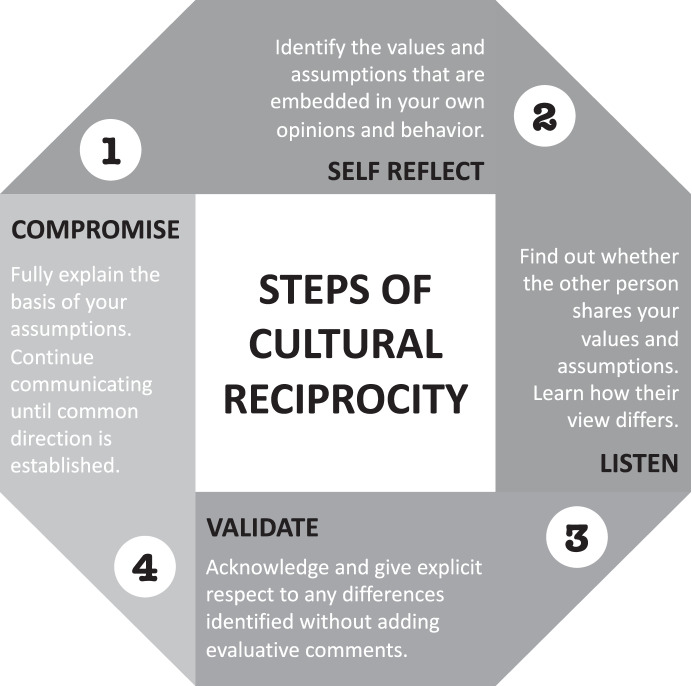
Steps of cultural reciprocity, adapted from Kalyanpur and Harry (1999)

Therefore, we introduce the steps of cultural reciprocity in the following sections to facilitate humble behaviorists’ adoption of IPP. Adapted from literature in the field of multicultural special education, cultural reciprocity is an ongoing process of interpersonal interaction and negotiation that demands individuals examine their own cultural biases and those of their profession (Kalyanpur & Harry, 1999). Cultural reciprocity aligns with the attitude of cultural humility and contradicts the attitude of disciplinary centrism. It organizes a set of actions—self-reflect, listen, validate, and compromise (see Fig. 3)—that humble behaviorists can engage in as interprofessional collaborators. While they are referred to as steps, the cultural reciprocity actions are neither linear nor finite; they are ever present and recursive. To some extent, however, validating and compromising rely on self-reflection and listening. We provide multiple exemplars of questions and responses that align with the steps of cultural reciprocity in Table 1.

**Table 1:** Sample questions and statements that align with the steps of cultural reciprocity

**Self-Reflect**	
Is disciplinary centrism influencing my opinion and behavior?	
Am I practicing within my scope of practice?	
Am I honest about my scope of competence?	
What belief, value, or assumption is motivating my behavior?	
What assumptions have I made about my colleagues’ knowledge and skills?	
Is our disagreement related to cultural differences?	
What do I value most? Why?	
Does my behavior align with my values?	
What stereotypes am I relying on?	
Why do I care so much about this?	
What about this makes me uncomfortable?	
Do I think my culture is superior? Why?	
Am I demonstrating humility or hubris?	
Do I think I am better than others because of my abilities?	
**Listen**	
How do you explain that?	
Help me understand what you mean by…	
What would you recommend in this situation?	
Where can I read more about that?	
I’m still a little confused. Can you explain it again please?	
Thank you for teaching me about your perspective.	
Oh. I totally misunderstood. Thanks for correcting me.	
I am open to being persuaded. Tell me more.	
Let me try to summarize how I understand what you said.	
**Validate**	
This is an area in which our training differs.	
I appreciate you explaining it to me.	
That makes a lot of sense.	
Your contributions were vital to this solution. Thanks.	
Both approaches have merit.	
I see. You believe…	
You do _____ very well.	
I haven’t thought about it like that.	
Thank you for sharing your perspective.	
**Compromise**	
An alternative perspective is…	
I understand that differently.	
Both are good ideas. How should we decide which to choose?	
There is another option to consider.	
I’m not convinced yet, but I’m open to hearing more.	
Let’s evaluate the evidence and make a decision together.	
I’m willing to compromise as long as we use progress monitoring data to evaluate the decision.	
I think you have changed my mind about that.	

### Self-Reflect

Working and interacting effectively with people who think differently begins with self-reflection, or the act of identifying personal biases and assumptions about others’ thoughts, beliefs, and practices. In addition to relying on the best available evidence, behavior analysts also select treatment goals in consideration of values—their own, those of the client and/or caregivers, and in the context of the macro and micro culture (Rakos, 1983). Degrees and certifications in behavior analysis do not remove the human tendencies of personal and professional biases of scientists. Contingencies governing the education, training, and professional practice of behavior analysis shape our beliefs, values, knowledge, and actions. Although many behaviorists continue to do so (e.g., Brandon, 2021), it is counterproductive to deny the existence of bias in the application of behavior science.

To initiate and attempt self-reflection, behavior analysts must acknowledge their own biases, to include any preference for prioritizing goals that promote a client’s success within the context of larger society over programs most relevant to the client’s own family or household. In absence of self-reflection of their own preferences and values, behavior analysts may (unintentionally) elect to assess and target behaviors considered socially valid by the standards of their own culture. They may write a treatment goal to increase a child’s initiations to adults, despite the behavior being considered disrespectful within the family’s cultural norms. Rather, when engaged in self-reflection, humble behaviorists recognize their knowledge limitations (including cultural beliefs) and seek input from clients and families and support from other professionals to design socially valid and sustainable interventions. For example, Couto de Carvalho et al. (2017) drew knowledge from other social science disciplines and used an ethnographic approach in their study of tagging cultures in Brazil. By spending time with several Brazilian tagging communities, the researchers were able to better operationalize *tagging* (similar to “graffiti”) and keep culture and implementation feasibility in mind when designing interventions.

Self-reflection also helps behavior analysts realize they can never become “competent” in another’s culture, which we suggest includes the culture of other disciplines (Wright, 2019). The only appropriate method of acquiring cultural understanding about another person is to interact with them. While doing so, humble behaviorists can examine the interlocking behavioral contingencies that exist within their own professional community between members of different disciplines that maintain and transmit unique and shared cultural practices (Couto, 2019). Awareness of interprofessional cultural dynamics can foster the engineering of contingencies to benefit the client and the team (Knapp et al., 2017). Self-reflection exposes the contingencies that impede successful interprofessional relationships. In an IPP context, humble behaviorists actively tact their biases and seek to understand how they might influence their collaborative behaviors (see sample Self-Reflection Questions in Table 1). For example, if a behavior analyst espouses the belief that behavior analysis is “the superior” science, their manner of speaking to their colleagues from an “inferior” discipline may be condescending, impatient, and dismissive. Likewise, if a behavior analyst recommends a specific treatment approach but the team chooses differently, they might regard their teammates as less intelligent or criticize their colleagues. Essentially, self-reflection is nothing more than observing one’s own behavior and pinpointing private events (i.e., attitudes, beliefs, assumptions, and values) that exert control over that behavior. While some assumptions need to be challenged (e.g., disciplinary centrism, ableism), not all are harmful. For instance, the behavior analytic values of *the learner is always right* and *data will decide* can lead to accepting and honoring team decisions and enhance the process by which the team operates. Self-reflection establishes the operations necessary to motivate behavior analysts to actively listen to their interprofessional colleagues and reduce the likelihood of practicing from a position of arrogance. Only from positions of humility can true collaboration occur.

### Listen

After reflecting on their own biases and assumptions and those of their professional culture, humble behaviorists seek to learn about the values and beliefs of their colleagues. This requires listening, really listening. The goal of listening is to understand the contingencies governing the behavior of others and the history of antecedents and consequences that may be maintaining differing opinions and recommendations. Humble behaviorists show respect for diversity of thought by inviting and allowing conversations about plausible approaches to solving the problem at hand. They should ask sincere questions to encourage their colleagues’ explanations and offer affirming statements to show they are actively listening (see Listening Statements in Table 1). Statements such as, “Help me understand what you mean” and “I am open to being persuaded” imply a respect for diversity of thought that can facilitate productive conversations. Conversely, authoritative statements such as, “No. We need to do it this way” or blanketly dismissive and judgmental comments such as, “That’s not evidence-based” threaten the establishment and maintenance of interprofessional relationships. One objective of collaborating with other professionals should be to endear them to the possibilities of the science through positive interactions, rather than attempting to convert one’s colleagues to behavior analysis through shame and coercion.

Much of our personal and professional development comes from interactions with others, in which story sharing and collective discussions are efficient transmitters of cultural knowledge (Maggio, 2014; McCabe, 1997). Imagine a scenario in which a colleague shared information about a nearby horseback riding therapeutic program with a family of a child with autism. As part of that team, a behavior analyst who is unfamiliar with this approach could protest the suggestion or gather more information. A humble behaviorist will likely ask their colleague some non-confrontational questions (e.g., “I don’t know much about that program. I’m interested in learning more. What do you like about it?”) and then listen. Although an immediate reaction could be to argue against the recommendation, the behavior analyst’s limited awareness requires them to take the listening role. While engaged in the discussion, the behavior analyst learns more about an unfamiliar therapeutic approach, as well as a little something about their colleague’s professional cultural background. Listening is one of the critical ways in which behavior analysts can maintain an interpersonal stance that is open to opposing viewpoints. Continuous self-reflection helps to remind the behavior analyst that their colleagues’ recommendations are culturally grounded and warrant unconditional respect. Rather than reflexively disagreeing, it is best to seek an understanding of the cultural determinants of the recommendation. Together, self-reflection and listening allow behavior analysts to gather information about similarities and differences in the team’s values and assumptions. Rapport is built upon the values shared by the team and trust is determined by how well the team navigates their differences.

### Validate

Interprofessional colleagues need to trust that behavior analysts are not going to criticize them or argue with them. While the absence of those behaviors is good, trust is cemented through the active validation of colleagues’ opinions, behaviors, and recommendations, especially under conditions of extreme disagreement. There are likely many mutually held beliefs among teammates that can serve as establishing operations for effective collaboration. However, in any relationship, the management of conflicting views can be challenging. When differences in opinion exist, the ability to maintain civil, respectful, and reciprocal dialogue is essential. In the moment, it may be necessary to check and recheck one’s own biases and tip the scale toward listening more and talking less. Validation is not designed to justify an experience, nor is it meant to drive consensus. It is simply allowing, accepting, and respecting diversity—cultural diversity. The most powerful thing behavior analysts can do is to acknowledge explicitly the differences and disagreements without adding evaluative comments (see Validating Statements in Table 1). The opposite of explicit acknowledgment is to dismiss diversity of thought. If a behavior analyst responds to their colleague’s idea with, “That isn’t going to work,” their colleague will likely feel dismissed and disrespected. However, if the behavior analyst says, “Thank you for sharing your perspective” or “I haven’t thought about it like that” they may be more likely to feel like their contributions to the team are appreciated and be willing to continue the conversation. Humble behavior analysts can use validation to reinforce the team’s value that all opinions matter and to demonstrate they are committed to the team process itself.

Validation within the context of interprofessional collaboration also involves explicitly stating others’ contributions. This part may be challenging for behavior analysts because they tend to be solution-focused and efficient (e.g., parsimonious verbal behavior). In addition, behavior analysts’ rule-governed behavior of only recognizing and using behavior analytic models or approaches may service the function of pliance or truth by authority (Belisle, 2020; Hayes et al., 1998; Kissi et al., 2017). It takes time, perspective taking skills, and substantial self-assurance to give overt credit to others, but statements such as, “You do _____ very well” and “That makes a lot of sense” serve the goal of acknowledging the strengths of multiple perspectives while simultaneously priming others to be open to behavior analytic solutions. Although a history of rule-governed verbal behavior maintained by pliance can further reinforce disciplinary centrism (Belisle, 2020), professional (i.e., cultural) humility improves our psychological flexibility in the consideration of non-behavior analytic models or approaches. Behavior analysts can acquire humility. Putting hubris aside for the benefit of clients can enhance the productivity and process of interprofessional teams. Alternatively, to invalidate another professional’s experience and contributions threatens the likelihood of equal distribution of power in interprofessional teams. The practice of cultural validation will be an asset to teams, and when behavior analysts validate regularly, others’ perception of the science of behavior is likely to improve.

### Compromise

The final step of cultural reciprocity demands skillful negotiation to reach an agreement by adjusting opposing views, also referred to as compromise. Although there are many definitions of *compromise* (e.g., to make a dishonorable or shameful concession; Merriam-Webster, n.d.), it is used here to highlight a key concept in the revised BACB Ethics Code. “Behavior analysts address conflicts by compromising when possible and always prioritizing the best interest of the client” (2.10). We applaud this addition to the new code and wish to emphasize that sometimes compromising is the best course of action. Also of importance is that *compromise* is used in relation to intra- and interprofessional collaboration. In the BACB Ethics Code the sentence with the word compromise follows, “Behavior analysts collaborate with colleagues from their own and other professions in the best interest of clients and stakeholders” (BACB, 2020, 2.10).

Compromising behaviors are admittedly elusive, although necessary. After listening and inquiring of their colleagues’ cultural determinants, humble behaviorists fully explain the basis of their assumptions. As they do, they educate their colleagues on the cultural determinants of their own attitudes, beliefs, and recommendations, with the intention of finding points of agreement with their colleagues. Communication—emphasis on self-reflecting, listening, and validating—should continue until common direction is established. Listening for the purpose of learning and validating differences and sharing for the purpose of finding common ground should lead the team to identify priorities, reach an agreement, and develop a shared plan. Humble behaviorists plan for and adjust their recommendations based on evidence extracted from research, knowledge of client preferences, and the clinical expertise and ideas of their colleagues (Spencer et al., 2012). As Neuringer stated, “…all knowledge is provisional and that one’s most deeply held positions must continually be reconsidered” (1991, p. 1). This openness to others paves the way for compromise.

For behavior analysts with siloed training, collaboration and compromise may run counter to previously reinforced cultural beliefs and practices. While behavior analysts are willing to defer to physicians and related medical providers (e.g., ruling out medical causes of externalized behavior), they tend to dispute disciplines from social sciences with little regard for their empirical support. Behavior analysts who reduce or underestimate the contributions of other human service disciplines such as psychology or social work are less likely to modify their recommendations. In contrast, humble behaviorists recognize that diversity of thought from a team of experts is inevitable, making compromising a critical collaborative practice.

Behavior analysts might need to adjust the prioritization or timing of assessment or treatment approaches, alter the terms used to describe a behavior and its variables, or modify the phrasing of a goal to improve linguistic accessibility. However, we acknowledge that “not all nonbehavioral treatments…are created equal” (Brodhead, 2015, p. 71). Compromise does not require behavior analysts to abandon their science or their ethics. For example, a behavior analyst and an occupational therapist (OT) work together on a child’s team. The OT may recommend a sensory diet of brushing for the teacher to use, the purpose of which is to reduce the child’s problem behavior. Although the behavior analyst may be skeptical of that approach, they can create a plan for the teacher that ensures brushing is used before problem behavior occurs (i.e., as an abative operation) rather than accidentally applied contingently upon the occurrence of problem behavior. In addition to resources already available to behavior analysts working on interprofessional teams (e.g., Brodhead, 2015), the Compromising Statements in Table 1 are examples of ways to overtly signal a compromise for the best interest of the client and productivity of the team without forsaking behavior analysis. One of our favorites is, “I’m not yet convinced, but I’m open to hearing more.”

### Putting It All Together

Imagine that a behavior analyst on an interprofessional team oversees the provision of home-based behavior therapy services for a young child with autism. The family explores options for promoting the child’s social perception skills and consults the speech-language pathologist and the behavior analyst to help them decide what approach the team will use. After researching various options online and consulting the behavior analytic literature, the behavior analyst offers the family a list of interventions designed by other behavior analysts. Conversely, the speech-language pathologist recommends the use of a social perception program that the behavior analyst knows does not have a substantial amount of research to support its use. After the family vocalizes their preference for the program with minimal evidence based on its alignment with their context and values, the behavior analyst must consider their course of action. They could refuse to collaborate with the speech-language pathologist, saying, “I can’t take part in that. It is not scientific.” On the other hand, the behavior analyst could align their actions with Ethics Code 2.10 (BACB, 2020), interprofessional competencies (IPEC, 2016), standards for interprofessional collaboration (Bowman et al., 2021), and the evidence-based practice of behavior analysis (Slocum et al., 2014). After all, families prefer behavior analysts who actively collaborate with other service providers (Callahan et al., 2019; Chadwell et al., 2018; Monz et al., 2019). Ultimately, in their consideration of family values and preferences, clinical expertise, and knowledge of the best available evidence, the behavior analyst adopts a position of humble behaviorism. They unite with their teammates to provide “the most efficient and effective interprofessional care” (Bowman et al., 2021, p. 1) and enact the steps of cultural reciprocity.

First, the behavior analyst engages in deliberate self-reflection. This leads to an acknowledgement that their apprehension to the family’s selection was grounded in the assumption that only interventions designed by behavior analysts are evidence-based or effective. They recognize their bias in that they searched for program effectiveness research only in behavior analytic journals. Additionally, they realize that limited research support is not the same thing as ineffective. The humble behaviorist can tact these private events and connect them directly to their own behavior. The behavior analyst actively listens to learn from their colleague, which also serves to avoid contention among the team and promotes future collaborations. They continue to seek information about the program and learn why the speech-language pathologist recommends it. They should ask, “I’m a little confused. Can you explain it again please?” or “Where can I read more about that?” As the speech-language pathologist shares their experiences with the program, the behavior analyst validates their knowledge and opinions and explicitly states areas of agreement. For example, they might say, “I appreciate you explaining it to me” or “I haven’t thought about it like that.” After acknowledging and validating differences, the behavior analyst decides that an effective compromise is possible, saying, “I think you have changed my mind about that.” The behavior analyst identifies components of the program that are conceptually aligned with the science of behavior and suggests that the program’s effectiveness can be enhanced by the addition of preference assessments and the strategic arrangement of materials, social partners, and settings. They also offer to create a clear progress monitoring plan with explicit data collection procedures and operational definitions. Although the team did not choose the behavior analyst’s recommendation, the behavior analyst was humble enough to find a way to contribute to the approach in a way that will likely enhance the child’s social perception. In addition, through compromise, the speech-language pathologist and family can learn more about the science and practice of behavior analysis and its value to the team.

## Summary

Complex world problems require creative and multifaceted solutions. Humble behaviorism and IPP will scale our science and practice to meet the needs of the world and all who live in it. Child maltreatment prevention programs (Prinz et al., 2009), positive parenting (Biglan, 2015), and occupational safety improvements (Geller, 2001; Gravina et al., 2019) are just some of the accomplishments resulting from the practice of humble behaviorism. Development, implementation, and widespread adoption of behavior analytic interventions require the support of other disciplines (Biglan, 2009; Lehman & Geller, 2004). IPP serves a function for the humble behaviorist, enhancing behavioral interventions’ scalability and the potential to bring about meaningful improvement in consumers’ quality of life and desired outcomes (Starry, 2016; White et al., 2018). In truly collaborative teaming, no one party believes they have more to teach than learn from their colleagues and no one team member has more say or control in the development and implementation of interventions.

We believe that behavior analysts have genuine compassion for others’ wellbeing and an invested interest in the expansion of behavior analysis. Nonetheless, we must acknowledge that the field of behavior analysis is relatively new in comparison to many social sciences (e.g., psychology, social work). More likely than not, our scientific peers have been working on solutions much longer than we have. Thus, in joining our colleagues to combat ails of contemporary society, we must be prepared to prevent or own up to the mistakes we are likely to make as we learn new interprofessional skills. We must be ready to genuinely apologize and commit to doing better. Each event in our personal and professional history is a learning opportunity. “There’s no shame in being wrong, only in refusing to learn” (source unknown). From a position of cultural humility and not disciplinary centrism, behavior analysts will be able to self-reflect, listen, validate, and compromise—repertoires our society desperately needs.

Humble behaviorism will require us to embrace the practice of cultural reciprocity. Likewise, we encourage behavior analysis training programs and faculty to embrace IPE to better prepare the next generation of behavior analysts for interprofessional collaboration. Behavior analysts need to know the four pillars of IPP: teams and teamwork, roles and responsibilities, values and ethics, and communication (IPEC, 2016). Furthermore, supervisors and mentors of early career behavior analysts must prompt and reinforce humble behaviors, champion interprofessional collaboration, and provide interprofessional teaming opportunities (Critchfield & Reed, 2017).

Humble behaviorism requires a commitment to exercising cultural humility and engaging in cultural reciprocity with others. Humble behaviorism will help us find personal and professional satisfaction in the values shared with collaborators and capitalize on the individual strengths of diverse professions to amplify our impact. Extending Neuringer’s vision, when behavior analysts practice humility…

We will be included in efforts to create solutions that resolve or ameliorate large scale, global problems.

We will transfer our behavioral technology across disciplines, ensuring its survival and maximizing its reach.

We will truly act in collaboration with others to serve our world.

## Footnotes

1 9 out of 161 total ethics violation notices were submitted by colleagues between 2016 and 2017 (BACB, 2018).
